# Decoding the hypoxic tumor microenvironment in colorectal cancer for prognostic modeling and therapeutic target discovery

**DOI:** 10.3389/fimmu.2025.1651749

**Published:** 2025-08-26

**Authors:** Xiao-Cui Duan, Yan Zhou, Fan Feng, Hai-Bo Jiang, Mei-Lin Wang, Zhe Han, Hong-Fei Pang, Yu-Hang Liu, Heng-Zhe Jia, Meng- He, Hong-Pan Xu, Yuan-Yuan Wang

**Affiliations:** ^1^ Department of Cell Biology, Institute of Basic Medicine, Hebei Medical University, Shijiazhuang, Hebei, China; ^2^ Department of Gastrointestinal Disease Center, The First Hospital of Hebei Medical University, Shijiazhuang, Hebei, China; ^3^ Department of Breast and Thyroid Diagnosis Center, The First Hospital of Hebei Medical University, Shijiazhuang, Hebei, China; ^4^ Department of Health Management Center, The First Hospital of Hebei Medical University, Shijiazhuang, Hebei, China

**Keywords:** colorectal cancer, hypoxia, single-cell sequencing, prognostic model, TME

## Abstract

**Background:**

Hypoxia is a hallmark of the colorectal cancer (CRC) tumor microenvironment (TME) that drives malignant progression, chemoresistance, and immune evasion. However, the cellular heterogeneity underpinning hypoxic responses in CRC and its impact on prognosis remain incompletely understood.

**Methods:**

We integrated single-cell RNA sequencing data from 15 CRC samples (GSE166555 and GSE221575) to delineate hypoxic and normoxic cell populations and identify hypoxia-related genes (HRGs). Weighted gene co-expression network analysis (WGCNA) and enrichment profiling elucidated key biological processes associated with hypoxia. Cell–cell communication networks were inferred using CellChat, and transcription factor regulatory modules were reconstructed via SCENIC and GRNBoost2. A hypoxia-based prognostic signature was developed from unique H3 cluster genes using univariate Cox and Lasso regression on The Cancer Genome Atlas (TCGA; n = 606) and validated in GSE39582 (n = 579). Drug sensitivity correlations were derived from the GDSCv2 database. Finally, *in vitro* assays assessed the functional role of GIPC2, a model gene, in CRC cell lines.

**Results:**

Single-cell profiling uncovered eight distinct hypoxic clusters, with H3 exhibiting the highest hypoxia scores and enrichment in glycoprotein metabolic and angiogenesis pathways. The eight-gene prognostic model stratified patients into high- and low-risk groups with significantly different overall survival in both TCGA (P = 0.0026) and validation cohorts (P = 0.011). Drug analysis highlighted associations of model genes with PI3K/MTOR and apoptosis pathways. GIPC2 knockdown in LS180 and HT-29 cells markedly inhibited proliferation, migration, and invasion, while inducing apoptosis and reversing EMT phenotypes.

**Conclusions:**

We present a robust hypoxia-related gene signature that accurately predicts CRC patient prognosis and nominate GIPC2 as a potential biomarker and therapeutic target, offering new insights into hypoxia-driven CRC biology and personalized treatment strategies.

## Introduction

CRC is one of the leading causes of cancer-related mortality globally, ranking as the third most common cancer and the second-highest cause of cancer death (1). The prognosis of CRC remains poor, primarily due to challenges in early detection and the development of metastasis (2). A growing body of evidence underscores the significant role of the TME in influencing CRC progression and patient outcomes (3). The complexity of the TME, coupled with its influence on tumor cell behaviors and therapeutic responses, calls for the development of improved prognostic models that incorporate TME characteristics.

A hallmark feature of most solid tumors, including CRC, is the presence of a hypoxic microenvironment, which significantly alters tumor biology and contributes to the malignant phenotype (4). Hypoxia induces the accumulation of hypoxia-inducible factors (HIFs), which reprogram cellular metabolism, protein synthesis, and cell cycle progression, enabling tumor cells to survive and proliferate under low-oxygen conditions (5, 6). The “Warburg effect”, characterized by increased glucose uptake and glycolysis even in the presence of oxygen, is driven by HIF-1 and MYC, providing essential nutrients and energy for rapid cell division and genome replication (7).

Hypoxia contributes to chemotherapy and radiotherapy resistance by inducing the expression of multidrug resistance proteins and enhancing drug efflux (8, 9). Additionally, hypoxia promotes immune evasion, as it increases the expression of programmed death ligand-1 (PD-L1) on tumor cells, suppressing immune responses and facilitating immune escape (10). The metabolic reprogramming driven by hypoxia also affects immune cell function, particularly in natural killer (NK) cells, by altering their transcriptome and reducing their ability to produce key immune-modulatory factors (11). Furthermore, hypoxia plays a crucial role in remodeling the extracellular matrix (ECM), increasing its stiffness and fibrotic content, which in turn enhances tumor cell invasion and metastasis (12). Hypoxia-induced changes in the TME, including the accumulation of regulatory metabolites such as lactate and adenosine, further contribute to immune suppression and the promotion of tumor progression (13). These metabolic shifts, coupled with the selective pressure exerted by hypoxia, shape the evolutionary landscape of the tumor, driving molecular aberrations that favor tumor survival and immune evasion.

Despite the well-established role of hypoxia in CRC, the specific molecular mechanisms through which it influences prognosis and therapeutic resistance remain incompletely understood. Recent studies have highlighted the potential of HRGs as biomarkers for predicting CRC patient outcomes, particularly in the context of immunotherapy. However, a comprehensive understanding of HRGs interactions within the TME and their impact on CRC progression is still lacking.

This study aims to address this gap by integrating single-cell transcriptomic data to explore the role of HRGs in CRC. By analyzing the interactions between HRGs and CRC cells, we aim to identify novel molecular markers for prognostic modeling and develop a hypoxia-related gene signature to improve the accuracy of CRC prognosis. Ultimately, our goal is to enhance the current clinical strategies for CRC management by providing a more precise model for predicting patient outcomes and guiding therapeutic decisions.

## Materials and methods

### Transcriptomic data acquisition and processing

RNA expression data and corresponding clinical information of CRC, comprising a total of 606 cases, were extracted from the TCGA database to serve as the training set for model development. Furthermore, the GSE39582 dataset, containing 579 colorectal cancer microarray data, was obtained from the GEO database to act as the validation set, assessing the model’s robustness and accuracy. All of this data was converted to TPM format and log2 transformed for subsequent analysis.

### Acquisition and processing of single-cell sequencing data

We extracted 13 CRC samples from the GSE166555 dataset and 2 CRC tumor samples from the GSE221575 dataset in the GEO database, totaling 15 tumor samples. Data analysis was conducted using R software (version 4.1.3) with the Seurat package. For cell quality control, we set the mitochondrial content to be no more than 20%, the hematopoietic cell content to be no more than 3%, and established standards for cell UMI counts and gene counts ranging from 200 to 20,000 and from 200 to 5,000, respectively. We employed the “normalizedata”, “findcariablefeatures”, and “scaledata” functions from the Seurat package for data normalization, selection of 2000 highly variable genes, and data transformation. Batch effect correction was performed using the harmony method. Subsequently, we utilized UMAP and t-SNE techniques from the Seurat package for dimensionality reduction and the Louvain algorithm for clustering analysis. Ultimately, we identified differentially expressed genes between various clusters or cell types using the “findallmarkers” function, with filtering criteria including a p-value less than 0.05, log2FC greater than 0.25, and an expression proportion greater than 0.1.

### Identifying hypoxic cells

Ensure that Python 3.7.3 and R 4.1.3, along with the R packages “corrplot” and “mclust”, are installed before commencing data analysis. Utilize the pre-downloaded single-cell sequencing data for analysis under the auspices of the CHPF package, which integrates this information to predict cellular hypoxia status. Ultimately, by analyzing the predicted outcomes, you can identify high-confidence hypoxic and normoxic cells, and infer the hypoxia status of other cells. The hypoxia gene sets required for data analysis can be found at the following link: https://github.com/yihan1221/CHPF.

### Cell annotation analysis

In our study, we systematically profiled the cellular composition of tumor tissues using a targeted panel of cell-specific markers. Tumor cells were identified using epithelial markers “EPCAM”, “KRT18”, “KRT19”, and “CDH1”. The stromal compartment was characterized by fibroblast markers “DCN”, “THY1”, “COL1A1”, and “COL1A2”, endothelial markers “PECAM1”, “CLDN2”, “FLT1”, and “RAMP2”, and immune cells including T-cells marked by “CD3D”, “CD3E”, “CD3G”, and “TRAC”; natural killer (NK) cells by “NKG7”, “GNLY”, “NCAM1”, and “KLRD1”; B-cells by “CD79A”, “IGHM”, “IGHG3”, and “IGHA2”; and myeloid cells by “LYZ”, “MARCO”, “CD68”, and “FCGR3A”. Subsequently, tumor cells were isolated and subjected to clustering analysis to dissect the intratumoral heterogeneity. The resulting cellular clusters were visualized using UMAP and t-SNE for dimensionality reduction, and bar charts and heatmaps were generated to quantitatively and qualitatively assess the distribution and expression patterns of key markers, respectively.

### WGCNA analysis and enrichment analysis

We employed the WGCNA software package to identify gene modules associated with hypoxia group cells. This analysis facilitated the clustering of co-expressed genes that may be related to the hypoxic phenotype. To elucidate the biological significance of these gene modules, we utilized the clusterprofiler software package for a comprehensive gene enrichment analysis, specifically querying the Gene Ontology Biological Process (GOBP) and Kyoto Encyclopedia of Genes and Genomes (KEGG) databases. This step revealed the functional annotations and pathways significantly enriched among the genes within the H group modules. Furthermore, to visually map and interpret the enrichment results, we harnessed cytoscape software, particularly employing the “enrichmentmap” and “autoannotate” plugins for an intuitive representation and annotation of the functional categories and pathways.

### Analysis of cell-to-cell communication and copy number variation analysis

To explore the potential interactions between immune and tumor cells, we utilized the R package “cellchat” for cell-cell communication analysis. This package simulates the communication process by integrating ligands, receptors, and their auxiliary factors. By analyzing the receptors expressed by one cell type and the corresponding ligands expressed by another, we inferred the enriched receptor-ligand interactions between these two cell types.

### Single-cell transcriptional factor analysis

Using the “scenic” package, we conducted predictions of transcription factors for cell clusters H3 and N3, respectively. Subsequently, we employed the GRNboost2 software for the co-expression analysis of genes to construct a gene regulatory network. We utilized the degree measure to identify significant nodes within the network, extracting the top 1% of genes or transcription factors for further analysis.

### Constructing and validating a prognostic model based on genes unique to hypoxia group

We analyzed unique and significant transcription factors or genes from the H3 cell cluster. Initially, we used univariate Cox analysis to screen genes associated with prognosis, followed by model building with Lasso regression analysis. We then calculated the risk scores, using the median value as the cutoff threshold to divide patients into high and low-risk groups.

### Cell culture

Human CRC cell lines (LoVo, HT-29, SW480, LS180, SW620) and normal fetal human colon (FHC) cells were purchased from the ATCC and maintained in DMEM (Gibco) supplemented with 10% FBS (HyClone) and 1% penicillin/streptomycin at 37°C in a humidified 5% CO_2_ atmosphere.

### Quantitative real-time PCR

Total RNA was extracted from tissues or cultured cells using TRIzol reagent (Invitrogen) according to the manufacturer’s instructions. cDNA was synthesized from 1 μg RNA using the PrimeScript RT Reagent Kit (Takara). qRT-PCR was performed on a QuantStudio 5 Real-Time PCR System (Applied Biosystems) using SYBR Premix Ex Taq II (Takara). Primer sequences for GIPC2 and GAPDH (internal control) are listed in **Supplementary Table S1**. Cycling conditions were: 95°C for 30 s, followed by 40 cycles of 95°C for 5 s and 60°C for 30 s. Relative expression was calculated by the 2^-^ΔΔCt method.

### siRNA transfection

GIPC2‐targeting siRNA (si-GIPC2) and non-targeting control siRNA (si-NC) were purchased from GenePharma. Cells were seeded at 50% confluence in 6-well plates and transfected with 50 nM siRNA using Lipofectamine RNAiMAX (Invitrogen) in Opti-MEM (Gibco). After 6 h, medium was replaced with complete DMEM, and cells were harvested 48 h post-transfection for downstream experiments.

### Cell proliferation assay (CCK-8)

Transfected LS180 and HT-29 cells were seeded in 96-well plates at 2,000 cells/well in quintuplicate. At days 1, 2, 3 and 4, 10 μL of CCK-8 reagent (Dojindo) was added to each well and incubated for 2 h at 37°C. Absorbance at 450 nm was measured using a microplate reader (Bio-Rad).

### Apoptosis assay by flow cytometry

Forty-eight hours after siRNA transfection, LS180 cells were collected, washed twice in cold PBS, and stained with Annexin V–FITC and propidium iodide (PI) using the Annexin V-FITC Apoptosis Detection Kit (BD Biosciences) following the manufacturer’s protocol. Samples were analyzed on a BD FACSCanto II flow cytometer, and data were processed with FlowJo v10.

### Western blot analysis

Total protein was extracted with RIPA buffer (Beyotime) containing protease and phosphatase inhibitors (Roche). Protein concentration was determined by BCA assay (Pierce). Equal amounts (30 μg) of protein were separated by 10% SDS-PAGE and transferred to PVDF membranes (Millipore). After blocking with 5% non-fat milk in TBS-T for 1 h, membranes were incubated overnight at 4°C with primary antibodies against cleaved caspase-3, Bcl-2, E-cadherin, vimentin (Cell Signaling Technology), and β-actin (Sigma). HRP-conjugated secondary antibodies (Jackson ImmunoResearch) were applied for 1 h at room temperature. Bands were visualized using ECL substrate (Thermo) and quantified by ImageJ.

### Transwell migration and invasion assays

For migration assays, 5 × 10^4^ transfected LS180 cells in serum-free DMEM were seeded into the upper chamber of 8-μm Transwell inserts (Corning). For invasion assays, inserts were pre-coated with 50 μL Matrigel (BD Biosciences) diluted 1:8 in serum-free DMEM. The lower chamber contained DMEM with 10% FBS. After 24 h (migration) or 36 h (invasion) at 37°C, non-migrated/invaded cells on the upper surface were removed with a cotton swab. Cells on the lower surface were fixed with 4% paraformaldehyde for 15 min, stained with 0.1% crystal violet for 20 min, rinsed, and air-dried. Five random fields per insert were photographed under an inverted microscope (Olympus) and counted. All assays were performed in triplicate.

### Statistical analysis

All data processing, statistical analysis, and plotting were performed in the R 4.1.3 software environment. We assessed the correlation between two continuous variables by calculating the Pearson correlation coefficient. Categorical variables were compared using the chi-squared test, while comparisons of continuous variables were conducted using the Wilcoxon rank-sum test or the T-test. Cox regression models and Kaplan-Meier survival curve analysis for survival analysis were implemented using the survival package. Statistical significance was set at a P-value of less than 0.05.

## Results

### Comprehensive single-cell transcriptome profiling reveals key cellular populations and hypoxic microenvironment in CRC

**Figure 1:**
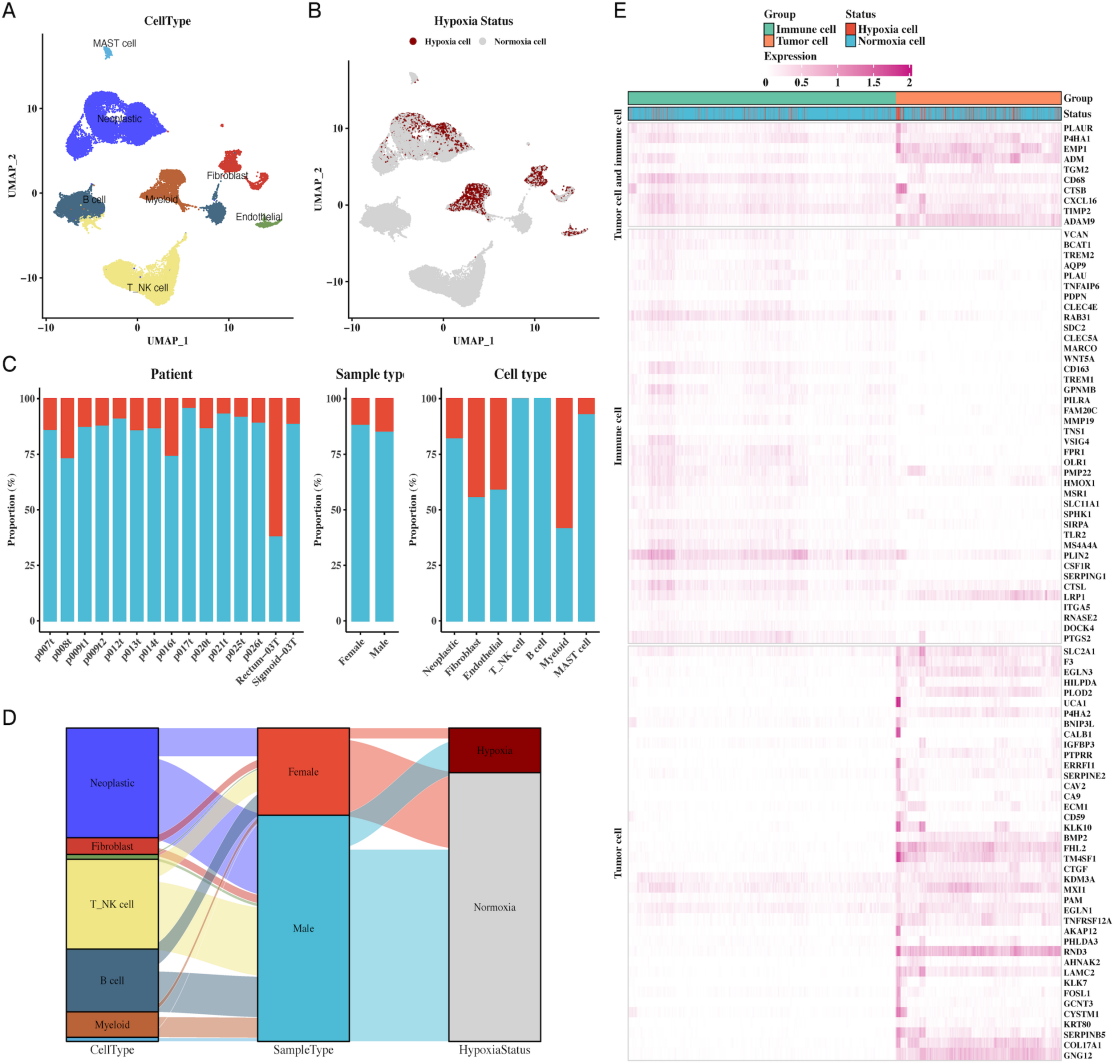
Single-cell transcriptomic profiling reveals cellular composition and hypoxic status in CRC. **(A)** UMAP plot of single-cell data showing distinct cell types in CRC, including endothelial cells, fibroblasts, myeloid cells, B cells, NK cells, and MAST cells, providing a comprehensive cellular atlas of CRC. **(B)** UMAP plot identifying hypoxic vs. non-hypoxic cells based on CHPF software, demonstrating the distribution of hypoxia across the single-cell dataset. **(C)** Bar chart of hypoxic and non-hypoxic cell proportions across CRC samples, tissue types, and cell types, highlighting variability in hypoxic conditions within the TME. **(D)** Sankey diagram illustrating the relationship between cell types, sample types, and hypoxic status, indicating the prevalence of hypoxia in different tumor and immune cell types. **(E)** Heatmap showing hypoxia marker expression in both tumor and immune cells, identifying key genes associated with hypoxia in these cellular populations.

We analyzed the cellular diversity and molecular characteristics of colorectal cancer (CRC) using the GSE166555 and GSE221575 datasets, which encompass 15 primary tumor samples. Using the UMAP algorithm for dimensionality reduction and clustering, we identified distinct cellular populations, including endothelial cells, fibroblasts, myeloid cells, B cells, NK cells, and MAST cells (**Figure 1A**). Hypoxic and non-hypoxic cell groups were classified using the “CHPF” package (**Figure 1B**), revealing that hypoxic cells were distributed across different tissue types and cellular compartments (**Figure 1C**). A Sankey diagram further demonstrated the association between cellular populations, tissue types, and hypoxia status, showing a strong correlation between immune cells and hypoxia (**Figure 1D**). Additionally, a heatmap of hypoxia markers highlighted their differential expression across tumor and immune cells (**Figure 1E**). **Supplementary Figure 1** provides additional insights into cell communication via the MIF signaling pathway.

### Immune cell subpopulations exhibit distinct hypoxic responses and interactions with tumor cells

**Figure 2:**
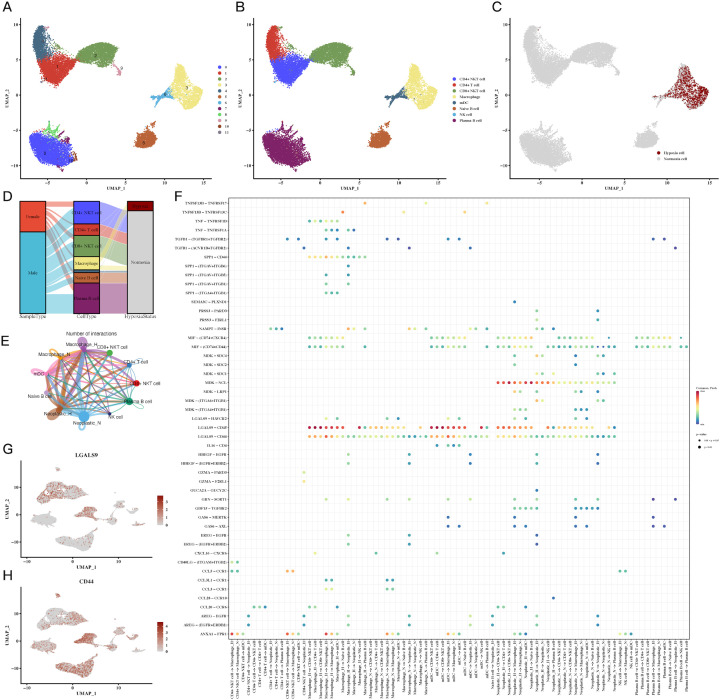
Immune cell subpopulations and their hypoxic status in CRC. **(A-C)** UMAP plots of immune cell subpopulations, showing distinct clustering and hypoxic status of immune cells, including macrophages and T cells, indicating immune cell adaptation to the hypoxic TME. **(D)** Sankey diagram of immune cell types, sample types, and hypoxic status, emphasizing the relationship between immune cells and the hypoxic environment. **(E, F)** Cell communication analysis between immune and tumor cells, showing key ligand-receptor interactions, particularly between LGALS9-CD44, indicating significant immune-tumor interactions in the hypoxic microenvironment. **(G, H)** Expression of LGALS9 and CD44 in immune cells and tumor cells, demonstrating their role in hypoxia-mediated immune modulation.

Further analysis of immune cell subpopulations revealed a close association between macrophages and hypoxia, particularly in the tumor microenvironment (**Figures 2A–C**). A Sankey diagram (**Figure 2D**) illustrated the interrelationship between immune cell types, tumor samples, and hypoxic status. To understand immune-tumor interactions, we employed CellChat for cell-cell communication analysis, identifying LGALS9-CD44 signaling as a significant pathway in CRC (**Figures 2E, F**). UMAP plots of LGALS9 and CD44 expression in both tumor and immune cells were visualized, confirming the importance of this signaling axis (**Figures 2G, H**).

### Tumor cell hypoxia subtypes reveal distinct molecular signatures and functional pathways

**Figure 3:**
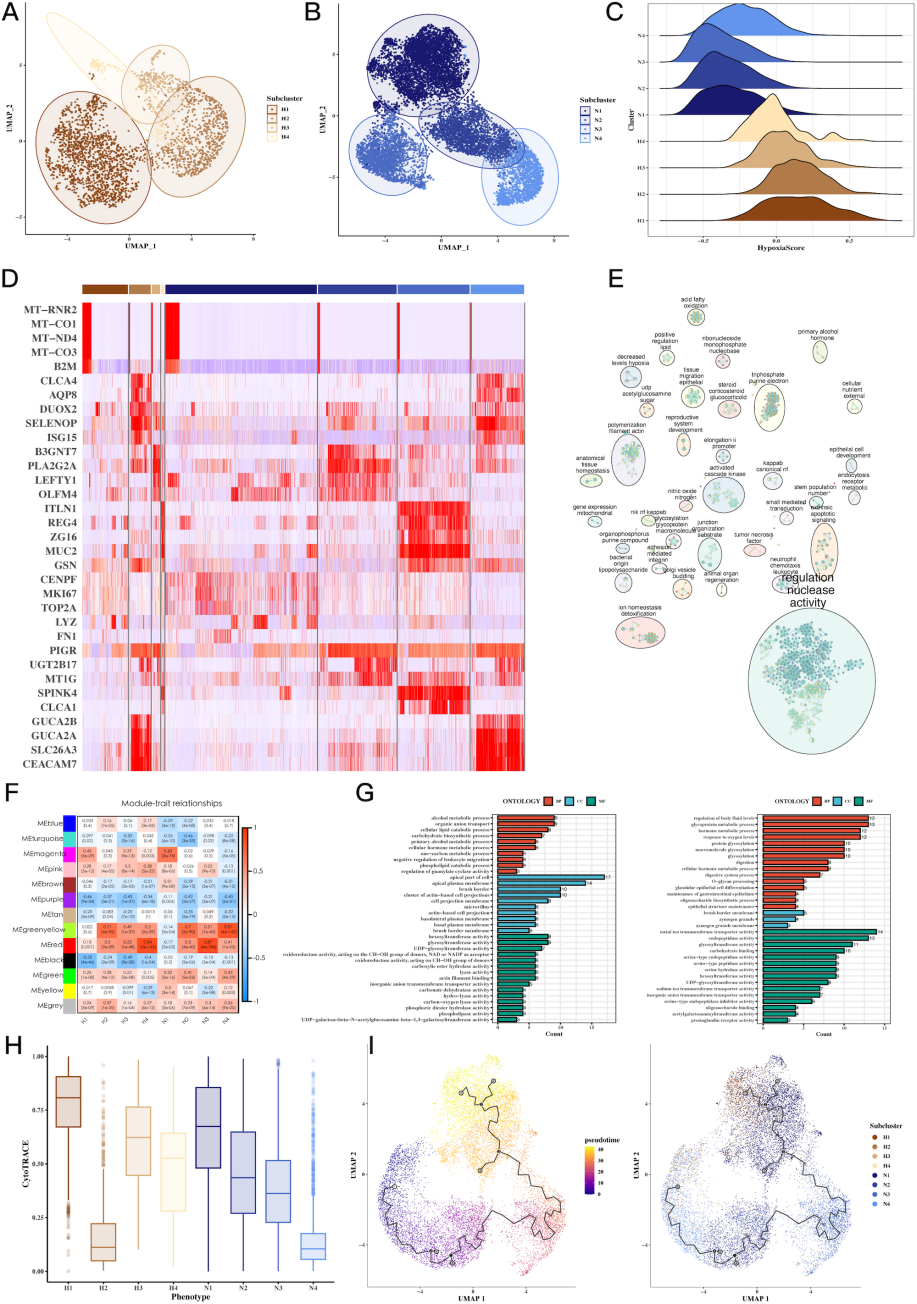
Tumor cell subgroup analysis reveals hypoxia-dependent tumor heterogeneity. **(A-C)** UMAP plots of hypoxic and non-hypoxic tumor cell clusters, along with ridge plots of hypoxia scores, revealing distinct hypoxic and non-hypoxic subpopulations within CRC tumors. **(D)** Heatmap of marker gene expression across tumor cell clusters, highlighting genes associated with hypoxia (e.g., MT-RNR2, MT-CO1), which distinguish hypoxic and non-hypoxic tumor subgroups. **(E)** GOBP enrichment analysis of marker genes from tumor subgroups, showing the biological processes enriched in hypoxic versus non-hypoxic tumor cells. **(F)** Heatmap showing the correlation between WGCNA gene modules and tumor cell subgroups, identifying specific gene modules associated with hypoxic tumor clusters. **(G)** GOBP enrichment analysis of WGCNA gene modules (green-yellow, red), revealing significant biological processes such as glycoprotein metabolism and oxygen response in hypoxic tumor cells. **(H)** CytoTRACE analysis of tumor cell differentiation potential, suggesting differential differentiation abilities between hypoxic and non-hypoxic tumor subgroups. **(I)** Pseudotime analysis of tumor cell differentiation using monocle3, showing the trajectory of tumor cell evolution and differentiation under hypoxic conditions.

Clustering of tumor cells based on hypoxic status identified four hypoxic clusters (H1, H2, H3, H4) and four non-hypoxic clusters (N1, N2, N3, N4). We characterized the molecular signatures of these clusters, with marker genes such as MT-RNR2, MT-CO1, MT-ND4, and MT-CO3B2M being enriched in hypoxic cells (**Figure 3D**). Hypoxia scores for each cluster were visualized, revealing higher scores in hypoxic subtypes (**Figures 3A–C**). GOBP enrichment analysis of the marker genes uncovered processes related to glycoprotein metabolism and cellular response to oxygen levels (**Figure 3E**). WGCNA analysis identified two key gene modules (green-yellow and red) associated with hypoxia, and further GO enrichment revealed significant involvement in metabolic and oxygen-sensing pathways (**Figures 3F, G**). Cytotrace analysis (**Figure 3H**) and pseudo-time analysis using Monocle3 (**Figure 3I**) confirmed the differentiation trajectories of these tumor subpopulations. **Supplementary Figures 2**, **3** provide additional details on hypoxia scores and Hallmark pathway enrichment in various tumor subgroups.

### Hypoxia-driven tumorigenic pathways, CNV alterations, and transcription factor networks

**Figure 4:**
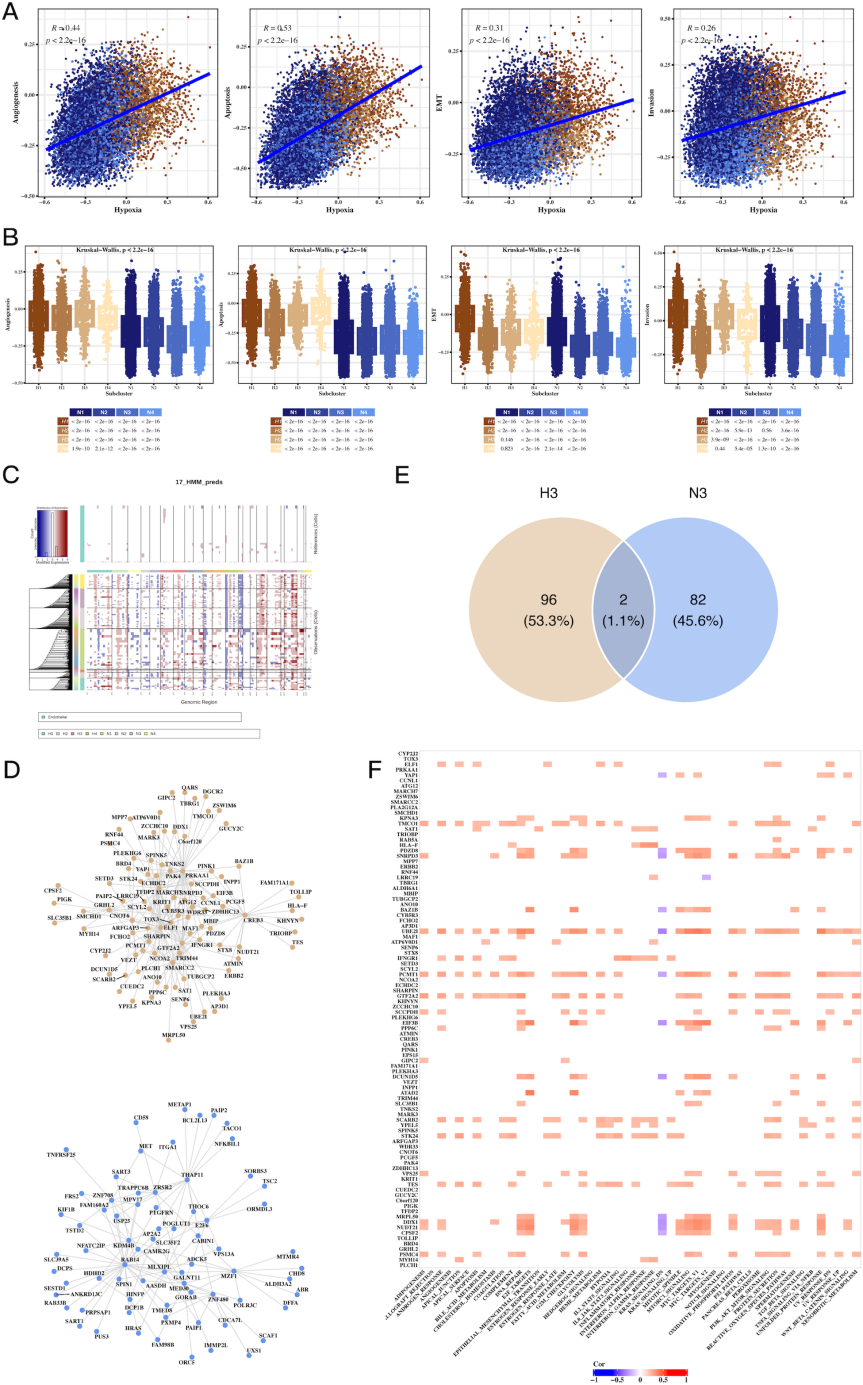
Hypoxia-associated pathways, CNV, and transcription factor alterations in CRC tumor cells. **(A)** Scatter plots showing the correlation between hypoxia scores and tumor progression signatures, including angiogenesis, apoptosis, EMT, and invasion, indicating hypoxia’s role in promoting aggressive tumor phenotypes. **(B)** Box plots comparing signature scores across different tumor cell clusters, highlighting significant differences between hypoxic and non-hypoxic subpopulations. **(C)** CNV analysis using inferCNV, identifying copy number variations in tumor cells compared to endothelial cells, revealing genomic alterations linked to hypoxic tumor cells. **(D)** Network graph of top transcription factors associated with hypoxic (H3) and non-hypoxic (N3) tumor clusters, highlighting key regulatory factors involved in tumor progression. **(E)** Venn diagram of overlapping transcription factors and genes between H3 and N3 clusters, emphasizing unique molecular signatures of hypoxic and non-hypoxic tumor cells. **(F)** Correlation heatmap of transcription factors and Hallmark pathways, showing significant associations between transcriptional regulators and key cancer-related pathways in hypoxic tumor cells.

We observed a strong correlation between hypoxia scores and tumor-related processes, including angiogenesis, apoptosis, epithelial-mesenchymal transition (EMT), and invasion (**Figure 4A**). Notably, H3 tumor cells exhibited the highest hypoxia scores, while N3 had the lowest (**Figure 4B**). CNV analysis revealed significant genomic alterations in tumor cells relative to endothelial cells (**Figure 4C**), with **Supplementary Figure 4** providing further insights into the CNV profiles across subgroups. Transcription factor analysis focused on H3 and N3 revealed unique regulatory networks, and a Venn diagram highlighted the top transcription factors specific to H3 (**Figures 4D, E**). Further correlation of these factors with Hallmark pathways identified key pathways involved in tumorigenesis (**Figure 4F**).

### Development and validation of a prognostic model based on hypoxia-related genes

**Figure 5:**
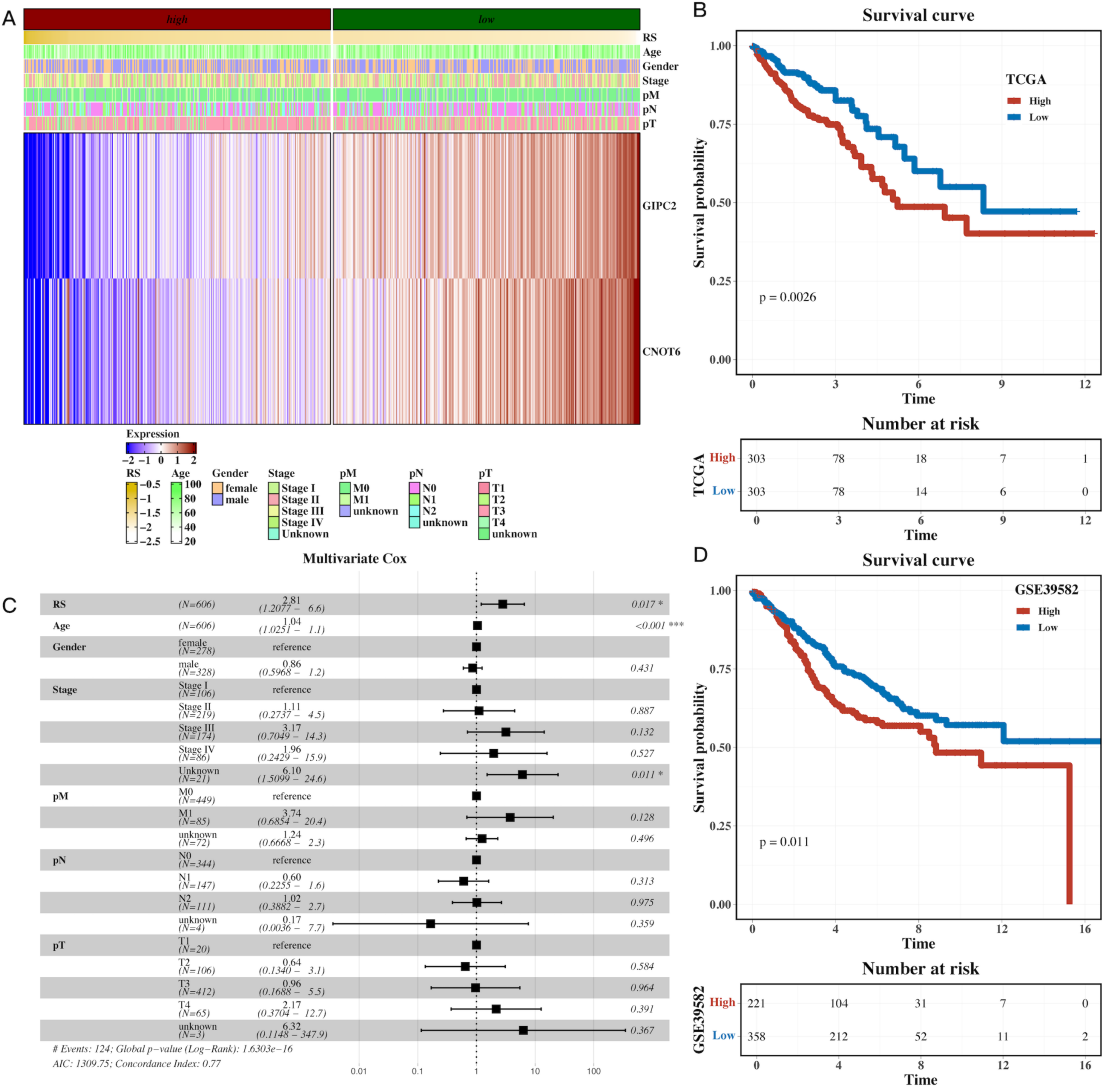
Prognostic model based on hypoxia-related genes identifies high-risk CRC patients. **(A)** Heatmap of model gene expression along with clinical indicators (e.g., age), demonstrating the predictive power of hypoxia-related genes for CRC prognosis. **(B)** Gene set variation analysis (GSVA) of TCGA dataset, showing pathway enrichment differences between high-risk and low-risk CRC groups based on the prognostic model. **(C)** Forest plot of multivariate Cox analysis, confirming the prognostic value of the risk scores derived from hypoxia-related genes, adjusted for clinical factors like age. **(D)** Survival analysis in the external cohort (GSE39582), showing that high-risk patients have significantly shorter overall survival compared to low-risk patients, validating the prognostic model’s robustness.

A univariate Cox regression analysis was performed to identify prognostic genes within the H3 hypoxic cluster. Using Lasso regression on the TCGA dataset, we constructed a prognostic model based on these hypoxia-associated genes (**Supplementary Figure 5**). Survival analysis demonstrated that high-risk patients, stratified by gene expression, had significantly shorter overall survival (OS) compared to the low-risk group (**Figures 5A,B**, P=0.0026). Multivariate Cox regression further confirmed the model’s robustness in predicting CRC prognosis, with clinical parameters also correlating with risk scores (**Figure 5C**). External validation using the GSE39582 dataset reinforced the prognostic value of the model, with high-risk patients exhibiting poorer survival (**Figure 5D**, P=0.011). These results highlight the prognostic potential of hypoxia-related genes in CRC.

### Drug sensitivity profiling and identification of therapeutic targets

**Figure 6:**
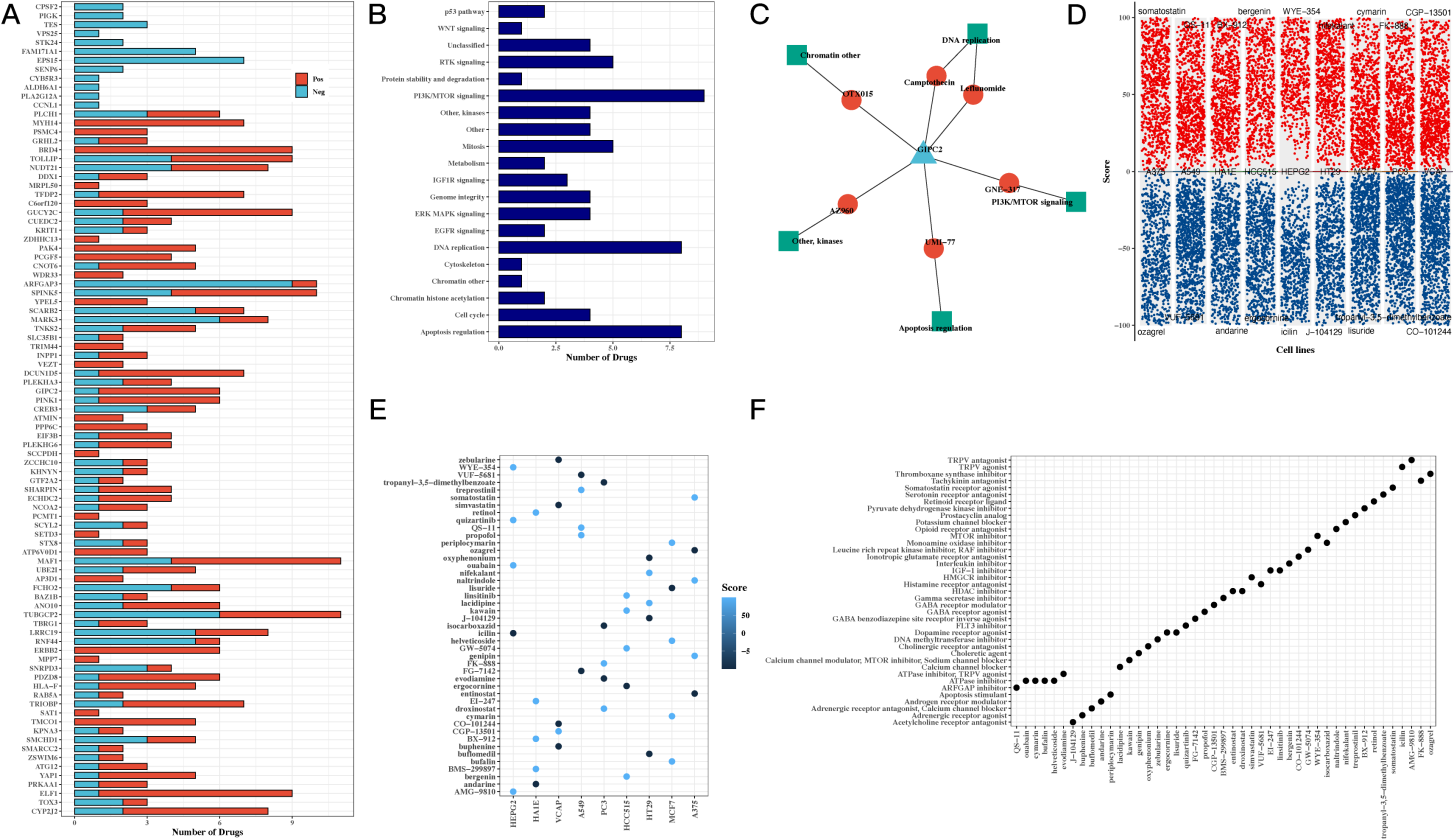
Drug sensitivity and pathway analysis reveals potential therapeutic targets for CRC. **(A)** Bar chart correlating transcription factors and drug sensitivity, showing the potential for targeting hypoxia-related genes in CRC treatment. **(B)** Bar chart of drug target pathways, identifying key pathways involved in the response to drugs, including PI3K/mTOR signaling and apoptosis regulation. **(C)** Network diagram of GIPC2, showing its interactions with drugs and target pathways, suggesting GIPC2 as a therapeutic target in CRC. **(D)** Volcano plots from CMap analysis, showing compound scores across various CRC cell lines, highlighting potential drugs that can target hypoxia-related pathways. **(E)** Bubble chart of top 5 compounds with the highest scores across CRC cell lines, providing insight into promising drug candidates. **(F)** Bubble chart summarizing the target pathways for the top 5 compounds, focusing on those with the highest efficacy in CRC cell lines.

To investigate the potential clinical applications of our findings, we conducted drug sensitivity analysis based on the GDSCv2 database. Genes and transcription factors from the H3 hypoxic cluster were correlated with drug sensitivity, revealing key compounds targeting PI3K/mTOR signaling, DNA replication, and apoptosis regulation (**Figures 6A, B**). A network diagram visualized the connections between the model gene GIPC2, associated drugs, and target pathways, suggesting that GIPC2 may influence apoptosis through its interaction with UMI-77 (**Figure 6C**). Volcano plots highlighted the ranking of compounds across various cell lines, and a summary of target pathways for each compound was presented (**Figures 6D–F**).

### GIPC2 promotes CRC cell proliferation and modulates key oncogenic processes

**Figure 7:**
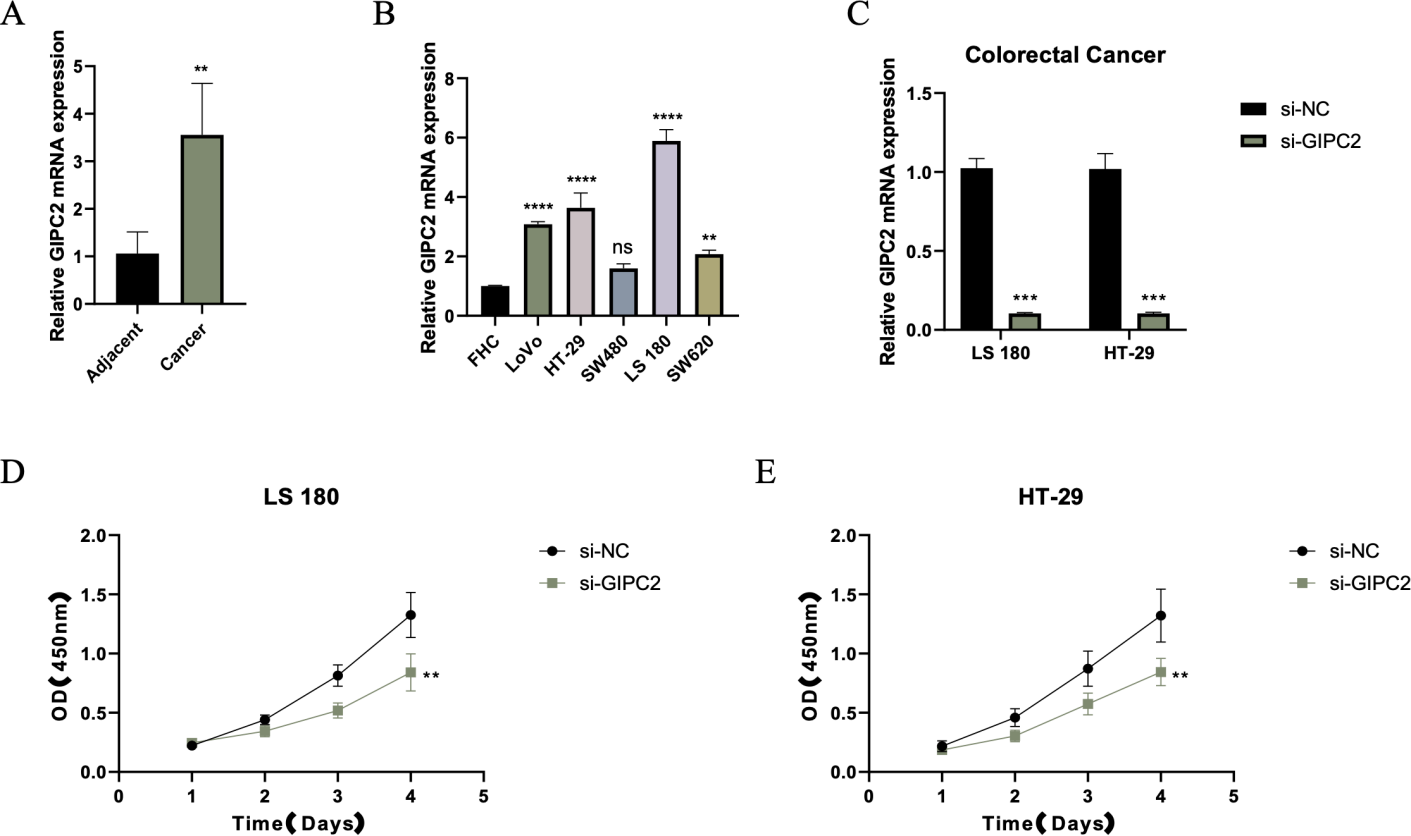
GIPC2 expression and its role in CRC cell proliferation and tumorigenesis. **(A)** Relative GIPC2 mRNA levels in paired CRC tissues and adjacent normal tissues, showing significant upregulation in tumor tissues. **(B)** GIPC2 expression in CRC cell lines compared to FHC control, revealing significant upregulation in SW480 and LoVo cell lines. **(C)** Efficiency of GIPC2 knockdown in LS180 and HT-29 cells, demonstrating effective silencing (>90%) after siRNA transfection. **(D, E)** CCK-8 assays of cell proliferation, showing that GIPC2 knockdown significantly reduces proliferation in LS180 **(D)** and HT-29 **(E)** cells over time (**P < 0.01; ***P < 0.001; ****P < 0.0001).

To explore the functional role of GIPC2 in CRC, we quantified its mRNA expression in paired CRC and adjacent normal tissues by qRT-PCR, finding a significant upregulation in tumor tissues (**Figure 7A**). Further analysis across CRC cell lines revealed that GIPC2 expression was markedly elevated in SW480 and LoVo cells (**Figure 7B**). Knockdown of GIPC2 in LS180 and HT-29 cells via siRNA significantly inhibited cell proliferation over a four-day period (**Figures 7C–E**), confirming GIPC2’s role as an oncogenic driver in CRC.

### GIPC2 knockdown induces apoptosis and inhibits EMT, migration, and invasion

**Figure 8:**
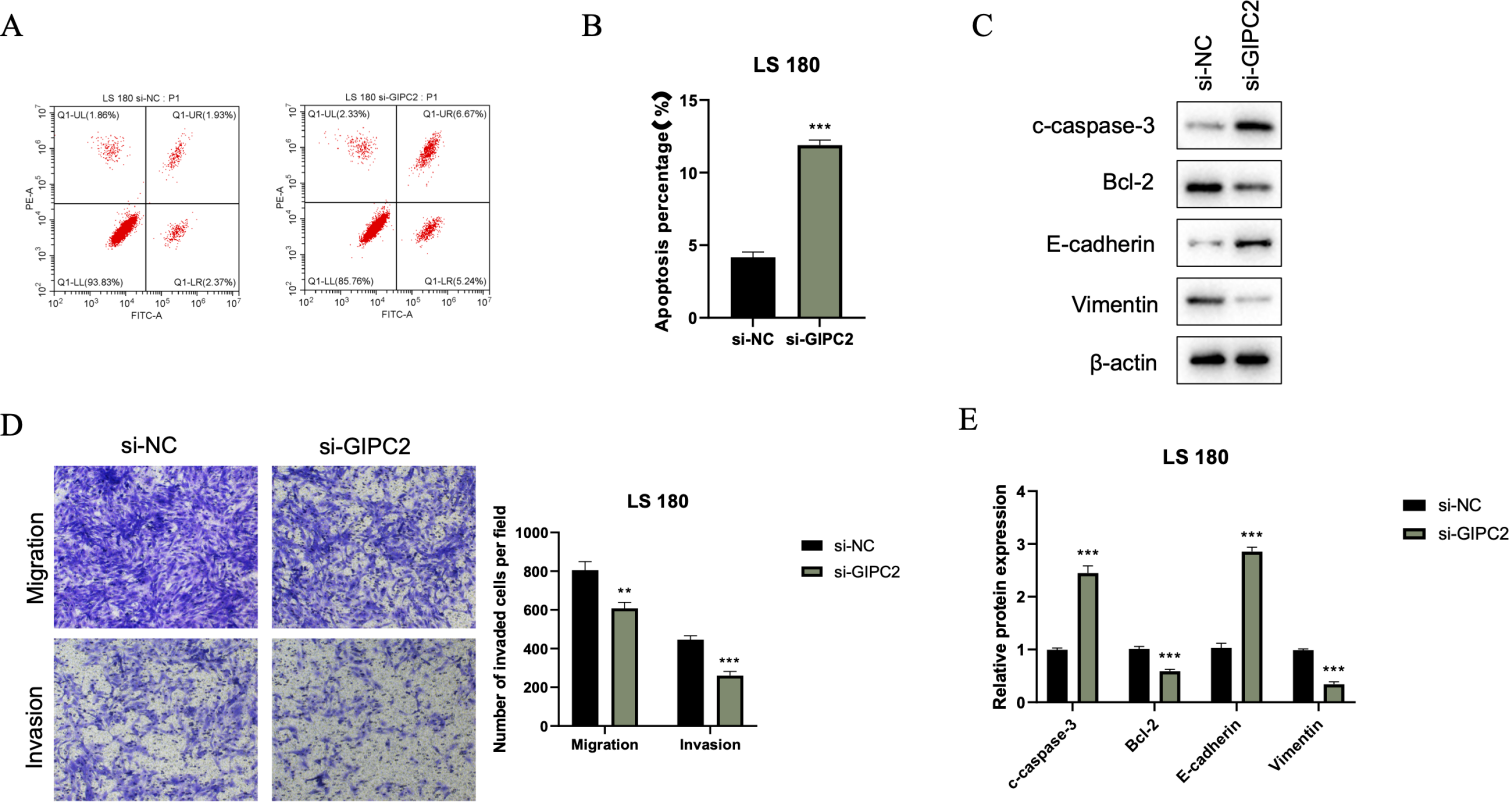
GIPC2 silencing promotes apoptosis, inhibits EMT, and reduces migration/invasion in CRC cells. **(A)** Flow cytometry analysis of Annexin V-FITC/PI staining in LS180 cells transfected with si-GIPC2 or si-NC, showing increased apoptosis following GIPC2 knockdown. **(B)** Quantification of total apoptotic cells (early + late) from three independent experiments, indicating a significant increase in apoptosis after GIPC2 silencing (**P < 0.01; ***P < 0.001). **(C)** Western blot analysis of cleaved caspase-3, Bcl-2, E-cadherin, and vimentin, showing activation of apoptosis and inhibition of EMT in GIPC2-knockdown cells. **(D)** Transwell assay images showing migration (top) and invasion (bottom) of LS180 cells following GIPC2 knockdown, highlighting reduced invasive capacity. **(E)** Quantification of migrated and invaded cells from three independent Transwell experiments, demonstrating significantly reduced migration and invasion after GIPC2 silencing (**P < 0.01; ***P < 0.001).

We further investigated the impact of GIPC2 on CRC cell survival and motility. Flow cytometry analysis revealed that GIPC2 silencing significantly increased apoptosis in LS180 cells (**Figures 8A, B**). Western blot analysis confirmed the activation of apoptosis (elevated cleaved caspase-3) and reversal of EMT (elevated E-cadherin, reduced vimentin) in GIPC2 knockdown cells (**Figure 8C**). Transwell assays demonstrated that GIPC2 silencing impaired cell migration and invasion (**Figures 8D, E**). These results indicate that GIPC2 promotes CRC progression by suppressing apoptosis and facilitating EMT, migration, and invasion, underscoring its potential as a therapeutic target.

## Discussion

In this study, we conducted a comprehensive analysis of the TME in CRC, with a particular focus on HRGs and their influence on CRC prognosis and treatment response. Our findings contribute to the understanding of the complex interactions within the TME and propose a novel prognostic model that could potentially improve patient outcomes.

TME is defined as the complex network of cells and extracellular matrix components that surround and support the growth and progression of a tumor (14, 15). This includes various cell types such as immune cells, fibroblasts, endothelial cells, and cancer-associated fibroblasts, as well as the secreted factors, signaling molecules, and the physical structure of the extracellular matrix (16, 17). The TME plays a crucial role in tumor initiation, growth, metastasis, and response to therapy (18, 19). In CRC, the TME is particularly significant as it can influence the behavior of cancer cells and affect patient prognosis (20, 21). Hypoxia, or low oxygen levels, is a common characteristic of the CRC TME, which can lead to the stabilization and accumulation of HIF (22). These factors promote angiogenesis, glycolysis, and immune evasion, contributing to tumor aggressiveness and resistance to chemotherapy and radiation therapy (23). Additionally, the TME in CRC can modulate the immune response, with tumor-associated immune cells either suppressing or enhancing anti-tumor immunity (24). The interaction between CRC cells and the TME is dynamic, with reciprocal signaling (25). Understanding the TME in CRC is essential for developing targeted therapies and improving patient outcomes. Our research findings indicate that macrophages may be closely associated with the hypoxic state of CRC. Previous studies have demonstrated that tumor-associated macrophages (TAMs) play a regulatory role in tumorigenesis, progression, metastasis, angiogenesis, and chemoresistance. For instance, colony-stimulating factor-1 (CSF-1) can promote the malignant transformation of breast cancer by attracting macrophages. TAMs can also facilitate the intravasation and extravasation of tumor cells by releasing epidermal growth factor (EGF) and vascular endothelial growth factor (VEGF). The EGF secreted by TAMs promotes the intravasation of tumor cells into blood vessels, while VEGF triggers the disruption of the endothelial cell barrier by breaking intercellular adhesion junctions. Furthermore, TAMs regulate the epithelial-mesenchymal transition (EMT) process, affecting tumor metastasis, through the STAT/miR-506-3p/FoxQ1 signaling pathway and the TAT/miR-506-3p/FoxQ1 pathway, and promote the degradation of the extracellular matrix by secreting matrix metalloproteinases and C-C motif chemokine ligand 18 (CCL18).

Our research also reveals intriguing associations between LGALS9 and CD44 with the hypoxic state in CRC. A study by Huang et al. indicated that LGALS9 expression is generally reduced in CRC tissues and is correlated with a poorer prognosis. Concurrently, they also found a strong positive correlation between the expression of LGALS9 and CD137, and demonstrated through a mouse model that the role of LGALS9 in CRC may depend on the expression of CD137 (26). Another study showed that in KRAS mutant cells, LGALS9 acts as a lysosomal inhibitor, suppressing the fusion of autophagosomes with lysosomes, leading to the accumulation of autophagosomes, excessive swelling of lysosomes, and cell death, offering a potential therapeutic avenue for anti-tumor treatment of CRC (27). CD44 may be involved in the ferroptosis process of CRC, thereby affecting the prognosis of CRC (28). Other research has found that CD44s is highly expressed in mesenchymal cell lines, while CD44 is highly expressed in epithelial cell lines. Knockdown of CD44 leads to reduced levels of vimentin expression and significantly inhibits cell proliferation, migration, and invasion. In CRC patients, the survival rate of the mesenchymal group and the high CD44 status group is significantly lower than that of the epithelial group and the low CD44 status group. Multivariate analysis indicates that CD44 status is an independent prognostic factor, suggesting that the status of EMT and CD44 is a key prognostic factor, and the switch of CD44 isoforms may be a trigger for EMT in CRC (29). These studies explore the possible links between LGALS9 and CD44 and the poor prognosis of CRC from different perspectives. Our study, combining single-cell sequencing with the tumor immune microenvironment, reveals the potential link between them and the poor prognosis of CRC from the perspective of hypoxia. Of course, these results require further validation through molecular mechanism experiments.

An exciting and novel aspect of our study is the identification of GIPC2 as a potential oncogene in CRC. Our findings show that GIPC2 is upregulated in CRC tumor tissues, which correlates with increased cell proliferation, migration, and invasion, suggesting that it functions as an oncogenic driver in CRC. This aligns with findings from other cancers, including gastric and breast cancer, where GIPC2 promotes tumorigenesis (30, 31). In cancers like acute lymphoblastic leukemia (ALL), GIPC2 is silenced through promoter hypermethylation, suggesting an epigenetic mechanism of downregulation (32). In contrast, we observed no such epigenetic silencing in CRC, which may imply that GIPC2 regulation in CRC follows different molecular pathways, such as genetic mutations or post-translational modifications. This hypothesis is further supported by the identification of F74Y, G102E, and E216X mutations in GIPC2 in CRC, which could disrupt its functional domains and contribute to tumorigenesis. Although our study highlights the upregulation of GIPC2, the precise molecular mechanisms by which it promotes CRC progression remain unclear. Existing literature suggests that GIPC2 interacts with Frizzled receptors in the WNT signaling pathway, a known regulator of CRC progression, and may also influence the PI3K/AKT pathway, which regulates cell survival and migration (33). These interactions, if validated in CRC, could position GIPC2 as a central player in the hypoxic TME, further promoting tumor aggression and metastasis.

### Limitations and future directions

This study provides important insights into the role of hypoxia and HRGs in CRC; however, several limitations should be acknowledged. First, the single-cell analysis was based on 15 CRC samples, which may limit the generalizability of the findings. While we utilized two independent datasets, further validation with larger or additional public datasets would strengthen the conclusions. Second, the prognostic model requires validation in larger, multi-center clinical cohorts to confirm its clinical applicability. Third, although GIPC2 is identified as a potential oncogene, its molecular mechanisms—particularly under hypoxic conditions—remain to be elucidated, and experimental validation *in vivo* is warranted. Finally, the potential of targeting the hypoxic TME with immunotherapy remains unexplored; future work should focus on the interactions between GIPC2, immune cells, and the hypoxic microenvironment to identify novel therapeutic strategies.

## Conclusion

In conclusion, our study unveils the intricate role of hypoxia in the tumor microenvironment of CRC, highlighting its influence on tumor biology and patient outcomes. The hypoxia gene signature prognostic model we developed not only predicts CRC prognosis with robust accuracy but also identifies potential therapeutic targets, paving the way for personalized treatment strategies.

## Data Availability

The original contributions presented in the study are included in the article/**Supplementary Material**. Further inquiries can be directed to the corresponding authors.

## Supplementary material

Supplementary Figure 1 Cell communication patterns in CRC TME **(A)**. Heatmap of cell communication results showing interactions between immune and tumor cells in CRC, including both incoming and outgoing signals. **(B)** Heatmap illustrating MIF signaling pathway communication patterns, showing its role in immune modulation across different cell types.

Supplementary Figure 2 Tumor subgroup and hypoxia scores **(A)**. UMAP plot of tumor cell subgroups, demonstrating clustering based on hypoxic status. **(B)** UMAP plot of hypoxia scores, highlighting the distribution of hypoxic regions across tumor subgroups. **(C)** Boxplots of hypoxia scores for hypoxic tumor subgroups, revealing variability in hypoxia levels.

Supplementary Figure 3 Pathway enrichment in tumor subgroups. **(A)** Heatmap of GSVA scores for Hallmark pathways, showing pathway enrichment in various tumor subgroups, with a focus on hypoxia-related pathways.

Supplementary Figure 4 Model construction and validation **(A, B)**. LassoCox model-building results, showing the selection of key prognostic genes
