# Supplementary figures and images for: Decoding the hypoxic tumor microenvironment in colorectal cancer for prognostic modeling and therapeutic target discovery
Source: Front Immunol. 2025 Aug 26;16:1651749. doi: 10.3389/fimmu.2025.1651749 (PMC12417453; doi:10.3389/fimmu.2025.1651749)

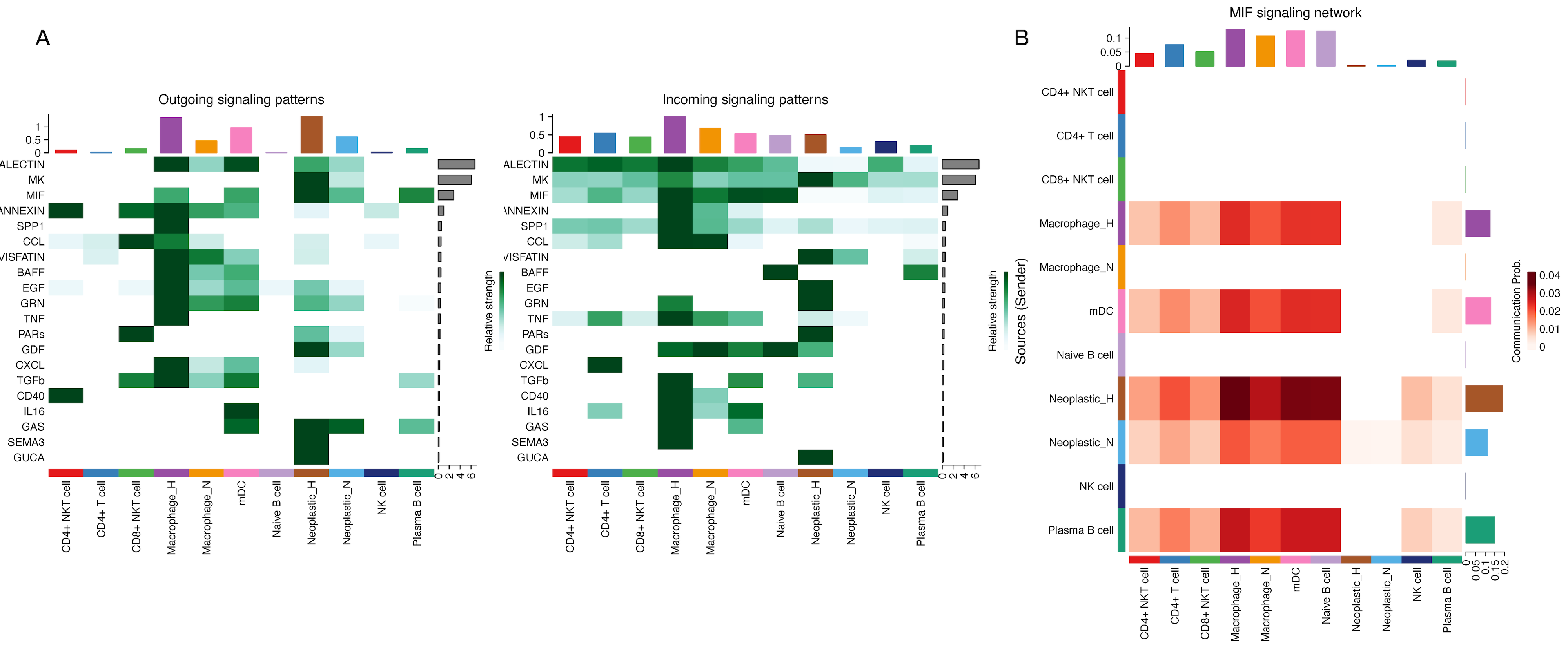

Supplement: Supplementary Figure 1 — Cell communication patterns in CRC TME (A). Heatmap of cell communication results showing interactions between immune and tumor cells in CRC, including both incoming and outgoing signals. (B) Heatmap illustrating MIF signaling pathway communication patterns, showing its role in immune modulation across different cell types. [file Image1.tif]

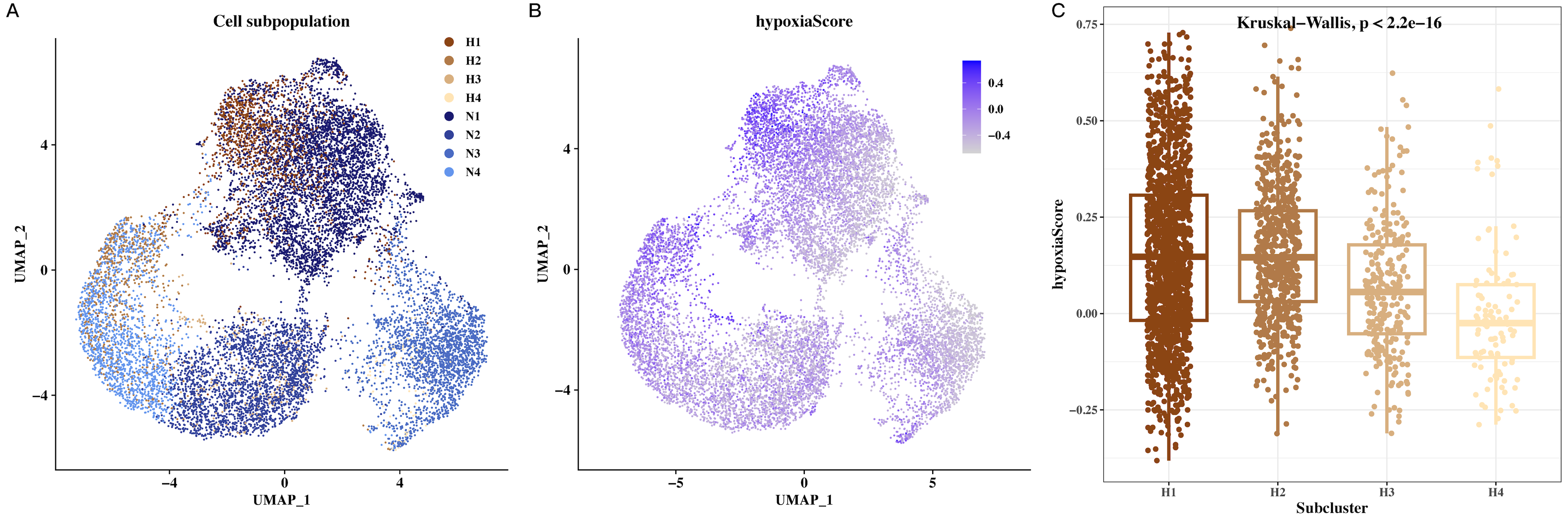

Supplement: Supplementary Figure 2 — Tumor subgroup and hypoxia scores (A). UMAP plot of tumor cell subgroups, demonstrating clustering based on hypoxic status. (B) UMAP plot of hypoxia scores, highlighting the distribution of hypoxic regions across tumor subgroups. (C) Boxplots of hypoxia scores for hypoxic tumor subgroups, revealing variability in hypoxia levels. [file Image2.tif]

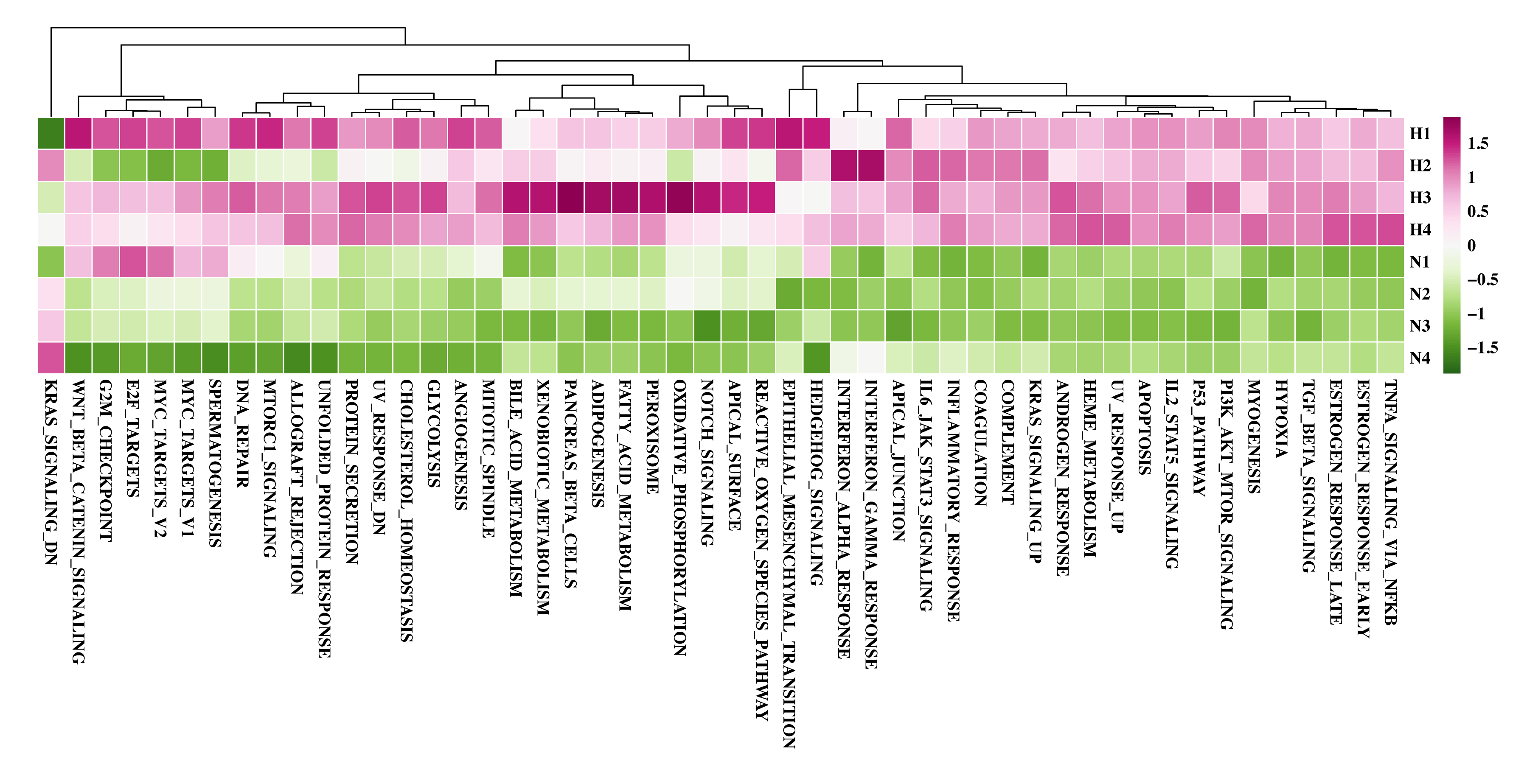

Supplement: Supplementary Figure 3 — Pathway enrichment in tumor subgroups. (A) Heatmap of GSVA scores for Hallmark pathways, showing pathway enrichment in various tumor subgroups, with a focus on hypoxia-related pathways. [file Image3.tif]

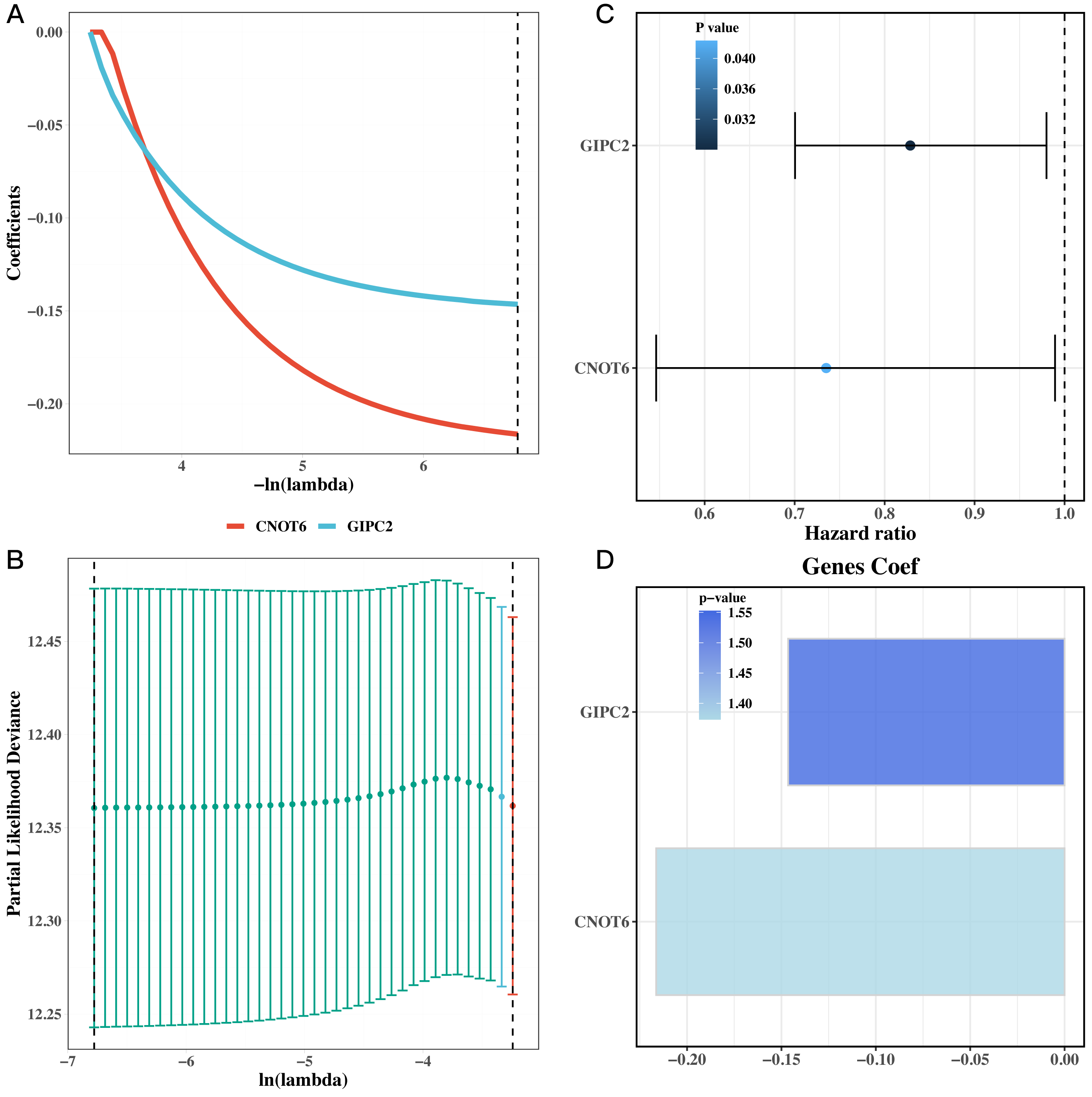

Supplement: Supplementary Figure 4 — Model construction and validation (A, B). LassoCox model-building results, showing the selection of key prognostic genes [file Image4.tif]

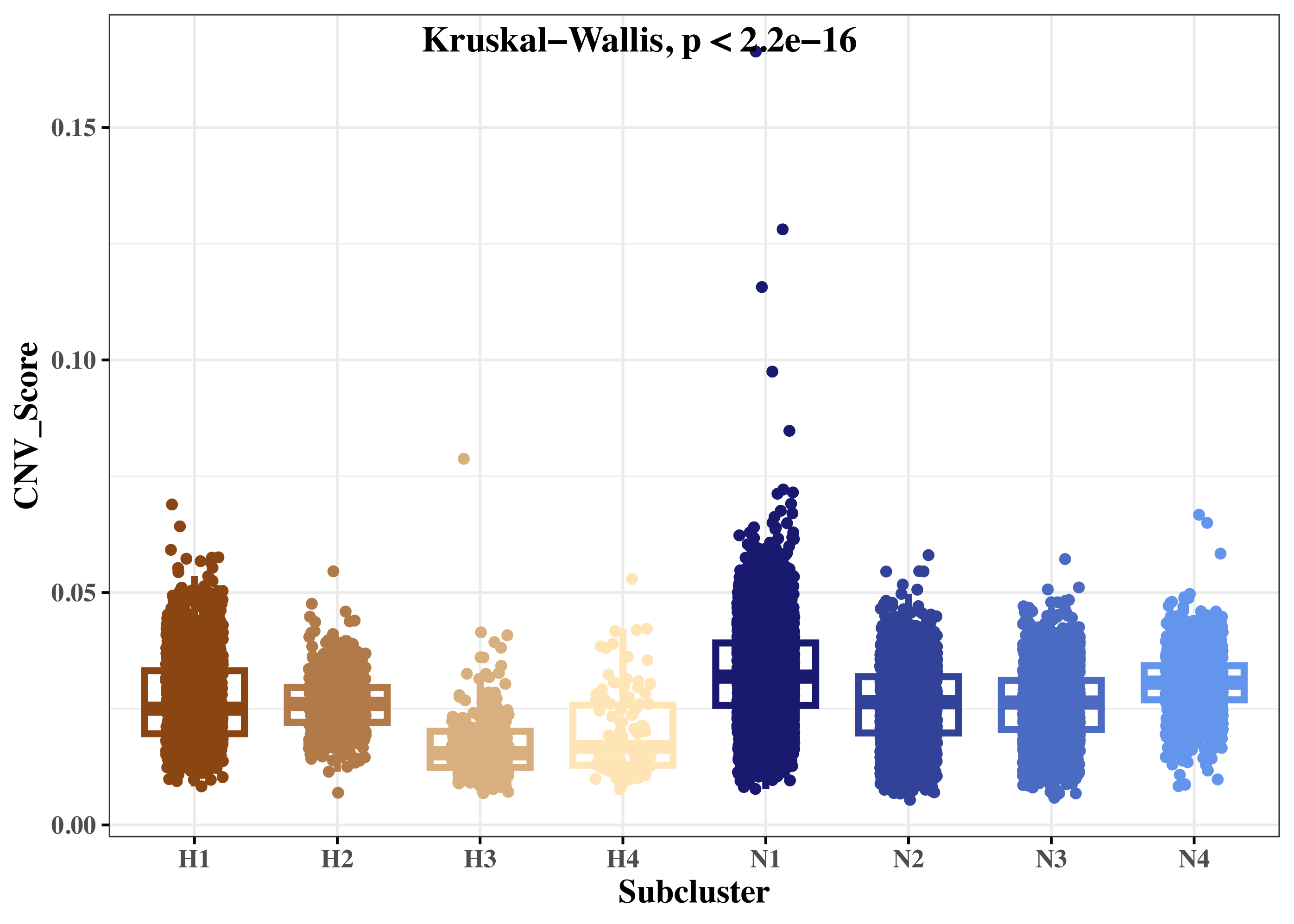

Supplement: Supplementary file 5 [file Image5.tif]
